# Tailoring the architecture of BiOI microspheres for enhanced photocatalytic wastewater purification

**DOI:** 10.3389/fchem.2026.1833474

**Published:** 2026-05-29

**Authors:** Yongxiang Ma, Junzhi Li, Shujie Liu, Liang Qiao, Xiuhua Zhu, Zhihua Zhang, Xiaoying Hu

**Affiliations:** 1 School of Materials Science and Engineering, Dalian Jiaotong University, Dalian, China; 2 School of Materials Science and Engineering, Key Laboratory of Materials Design and Quantum Simulation, Changchun University, Changchun, China

**Keywords:** BiOI hollow microspheres, controlled morphology, orthogonal experiment, photocatalytic degradation, wastewater purification

## Abstract

Bismuth oxyiodide (BiOI) features alternating [Bi_2_O_2_]^2+^ layers and double I^−^ interlayers, which forms an intrinsic electric field that enhances charge carrier transfer and facilitates the efficient separation of photogenerated electron-hole pairs. Owing to its exceptional visible-light absorption capability and high photocatalytic activity, BiOI has emerged as a promising candidate for environmental remediation. Herein, BiOI microsphere photocatalysts were fabricated *via* a facile solvothermal method, and various morphologies were obtained through different synthesis conditions. Among all BiOI catalysts synthesized in this work, the flower-like BiOI hollow microspheres (BiOI-6) exhibited superior dark adsorption and extremely fast photocatalytic degradation rates, with ultra-rapid kinetics and excellent stability. The rate constant k of BiOI-6 reached 0.130 min^-1^, which is 8.7 times higher than those of BiOI-5 and BiOI-7. This catalyst demonstrated a zeta potential of −34.20 mV, ensuring high dispersion stability, and possessed a bandgap energy of 2.07 eV as estimated using the Kubelka-Munk equation with a valence band position of 1.50 eV as determined by XPS. The high efficiency of the flower-like BiOI hollow microsphere catalyst was attributed to its large specific surface area, porous hollow structure, narrow bandgap energy, high hydrophilicity (water contact angle of ∼30°), and enhanced charge carrier separation efficiency. Based on these findings, BiOI shows significant potential in the development of efficient and stable photocatalysts for wastewater pollution control and purification.

## 1 Introduction

Semiconductor photocatalysis offers a promising solution to the issues caused by the rapid pace of global industrialization and economic growth, coupled with the increasing environmental pollution and energy shortages (Anwer et al., 2019; Kefeni and Mamba, 2020; Kim et al., 2024). This approach utilizes sunlight to degrade toxic pollutants into harmless CO_2_, H_2_O, and mineralized species (Das et al., 2024; Li S. et al., 2024; Li Y. et al., 2024; Wang et al., 2024); it provides unique advantages such as low energy consumption, cost-effectiveness, and minimal secondary pollution (Aljuaid et al., 2023; Fulzele et al., 2022; Kumar and Chanana, 2023; Zhang et al., 2025). Consequently, it has attracted significant research attention in recent years.

Bismuth (Bi)-based photocatalysts have attracted considerable research interest owing to their visible-light responsiveness, unique layered structure, and adjustable electronic bandgap (Feng et al., 2020; Huang et al., 2015; Liu et al., 2020; Zheng et al., 2024; Zhou et al., 2024). Furthermore, Bi is considered a “green metal” due to its unique position as one of the few naturally occurring heavy metals with low toxicity and negligible radioactivity, ensuring minimal environmental and biological impacts (Hassan et al., 2022; Xie, 2023). Bismuth oxyhalides (BiOX, X = Cl, Br, I), a novel type of 2D layered photocatalytic material with Sillén structure, are composed of positively charged [Bi_2_O_2_]^2+^ layers and negatively charged double halide ions aligned along the z-axis. Their combination creates an intrinsic electric field (IEF) (Liu and Wu, 2015). The IEF accelerates the transfer of charge carriers and the efficient separation of photogenerated electron–hole (e^−^/h^+^) pairs (Sun et al., 2023). By enhancing the utilization of visible light and improving the separation of e^−^/h^+^ pairs, morphology control has emerged as a promising strategy for developing efficient photocatalysts (Li et al., 2022; Liu et al., 2023; Wu et al., 2023).

In recent years, numerous efforts have been made to fabricate BiOX materials with various morphologies, such as nanowires (Zhao et al., 2021), nanobelts (Qu et al., 2018), nanosheets (Yuan et al., 2021), nanoparticles (Paengjun and Ogawa, 2021), nanoflowers (Yan et al., 2023), and hierarchical nanostructures (Zhang et al., 2022). In order to enhance their photocatalytic efficiency, He et al. reported that BiOBr nanosheets with predominantly exposed (001) or (010) facets were fabricated using a one-pot mannitol-assisted hydrothermal method (Li M. et al., 2024). Zhao et al. reported the successful synthesis of tree-like nanofiber TiO_2_/BiOI heterojunctions by growing BiOI on electrospun TiO_2_ using the solvothermal method (Zhang et al., 2024). Bismuth oxyiodide (BiOI) is characterized by an exceptional visible light response and exhibits superior photocatalytic activity under visible light exposure compared to BiOCl and BiOBr, rendering it a pivotal research focus (Di et al., 2014; Zhu et al., 2022). Nevertheless, previously synthesized BiOI particles showed unsatisfactory photocatalytic performance under visible light, severely limiting their practical application. Novel methodologies are required to enhance the photocatalytic activity of BiOI.

In this study, BiOI photocatalysts were synthesized *via* a solvothermal method, and the optimal flower-like hollow microsphere structure was achieved through precise morphological control. Various characterization techniques, including X-ray powder diffraction (XRD), scanning electron microscopy (SEM), transmission electron microscopy (TEM), X-ray photoelectron spectroscopy (XPS), UV-vis diffuse reflectance spectroscopy (UV-vis DRS), wettability tests, electrochemical analysis, Fourier transform infrared (FTIR) spectra, and Raman spectra provided detailed insights into the structural, morphological and chemical features that enhanced the photocatalytic activity of the BiOI catalysts. The experimental results show that this catalyst has a large specific surface area, a hollow porous structure and a narrow bandgap energy. As a result, the flower-like hollow microsphere catalyst demonstrates superior dark adsorption capacity, remarkably fast degradation kinetics, and outstanding stability.

## 2 Experimental section

### 2.1 Synthesis of BiOI

The orthogonal experimental design was employed to minimize the number of trials, identify optimal conditions, and determine the degree to which critical factors influence performance (Avila-López et al., 2022; Sun et al., 2022). In this study, an L9 (3^4^) orthogonal table was used to design a four-factor, three-level BiOI synthesis experiment (with the fourth factor being the control group). The orthogonal experiment procedures are detailed in Supplementary Table S1.

The preparation flow for the BiOI synthesis is described below. First, 1.455 g (0.003 mol) of Bi(NO_3_)_3_·5H_2_O was dissolved in 20 mL of, E.G.,/H_2_O mixtures in varying proportions, with the obtained solution denoted as Solution A. Subsequently, 0.498 g (0.003 mol) of KI was dissolved in 20 mL of, E.G.,/H_2_O mixtures in different ratios, with the obtained solution denoted as Solution B. Then, Solution A was added dropwise to Solution B under vigorous stirring (600 r/min) at room temperature for 30 min to form a homogeneous suspension. The resulting suspension was then transferred to a polytetrafluoroethylene-lined solvothermal reactor and subjected to a solvothermal reaction at temperatures of 100, 120, or 140 °C for periods of 6, 8, or 10 h. After the reaction, the reactor was cooled to room temperature. The precipitated sample was washed multiple times with deionized water and ethanol and then dried at 60 °C for 12 h to obtain the final BiOI samples, designated as BiOI-X (where X denotes the position in the synthesis sequence).

## 3 Results and discussion

The degradation rate of Rhodamine B (Rh B) after 5 min of visible light irradiation was set as the reference for the evaluation. This choice was based on the observation that the optimal BiOI-6 catalyst could degrade 97.15% of Rh B under visible light irradiation for 5 min, and the final degradation rates of the other samples were consistent with those at 5 min. The detailed experimental procedure is illustrated in Table 1. Variance analysis revealed that the main factor affecting the BiOI removal performance was the solvent, with the range of pure, E.G., and, E.G.,/H_2_O (1:1 volume ratio) as the solvent being 186.15, approximately five times that of the temperature factor. The range of the control group was greater than that of the time factor, indicating that the selected time had negligible influence during the BiOI synthesis. Based on the orthogonal experiment analysis and degradation efficiencies, the optimal synthesis conditions for BiOI were identified as follows: pure, E.G., as the solvent, a reaction temperature of 120 °C, and a reaction time of 10 h, with the solvent type being the critical factor.

**TABLE 1:** Results of orthogonal experiment.

Sequence	Factor				Experimental results (degradation rate, %)
	A Temperature (°C)	B Time (h)	C Volume ratio of, E.G., to H_2_O	D Control group	
1	100	6	4:0	1	86.40
2	100	8	3:1	2	32.80
3	100	10	1:1	3	25.45
4	120	6	3:1	3	49.45
5	120	8	1:1	1	34.20
6	120	10	4:0	2	97.15
7	140	6	1:1	2	25.10
8	140	8	4:0	3	87.35
9	140	10	3:1	1	31.95
*K*_1_	144.65	160.95	270.90	152.55	​
*K*_2_	180.80	154.35	114.20	155.05	​
*K*_3_	144.40	154.55	84.75	162.25	​
Range	36.40	6.60	186.15	9.70	​
Order of influence	C >> a > B				​
Optimal level	A_2_	​	C_1_	​	​
Optimal combination	A_2_C_1_				​

**FIGURE 1:**
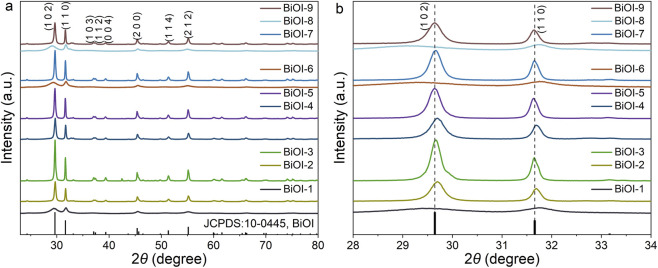
**(a,b)** XRD patterns of BiOI-1–BiOI-9 samples.

XRD analysis was conducted to investigate the crystal properties of various BiOI samples. Figure 1a shows the XRD patterns of the BiOI-X samples. All primary diffraction peaks matched the standard JCPDS card No. 10-0445 (BiOI, *P*4/*nmm* unit cell parameters, *a* = *b* = 3.994 Å, *c* = 9.149 Å, *α* = *β* = *γ* = 90°) without any impurity peaks. This indicates that the phase of the BiOI powder samples synthesized under different conditions remained unchanged, and the obtained materials were single-phase BiOI crystals. The diffraction peaks at 2*θ* = 24.292°, 29.645°, 31.657°, 37.057°, 37.392°, 39.365°, 45.377°, 51.345°, and 55.150° corresponded to the (101), (102), (110), (103), (112), (004), (200), (114), and (212) crystal planes of the BiOI samples, respectively. Among them, the strong and sharp diffraction peaks of the BiOI-3, BiOI-5, and BiOI-7 samples indicated their good crystallinity. However, increasing the, E.G., proportion resulted in smoother diffraction peaks and the disappearance of certain lower-intensity peaks, indicating a gradual decrease in crystallinity. According to the Debye–Scherrer equation, the full width at half-maximum (FWHM) showed a gradual increase, indicating a corresponding decrease in the grain size of the synthesized samples (Aghdam et al., 2018). The crystallinity of BiOI was found to decrease with increasing, E.G., content in the solvent. However, this did not result in any change in the crystal phase structure. The XRD pattern of the BiOI samples in the 2θ range from 27° to 34° shows that, with increasing proportions of, E.G., the (102) and (110) diffraction peaks shifted slightly to higher angles (Figure 1b). This may be due to lattice defects-induced lattice shrinkage present in the samples, which can influence the lattice parameters, leading to a shift of the diffraction peaks to higher angles (Arif et al., 2022).

**FIGURE 2:**
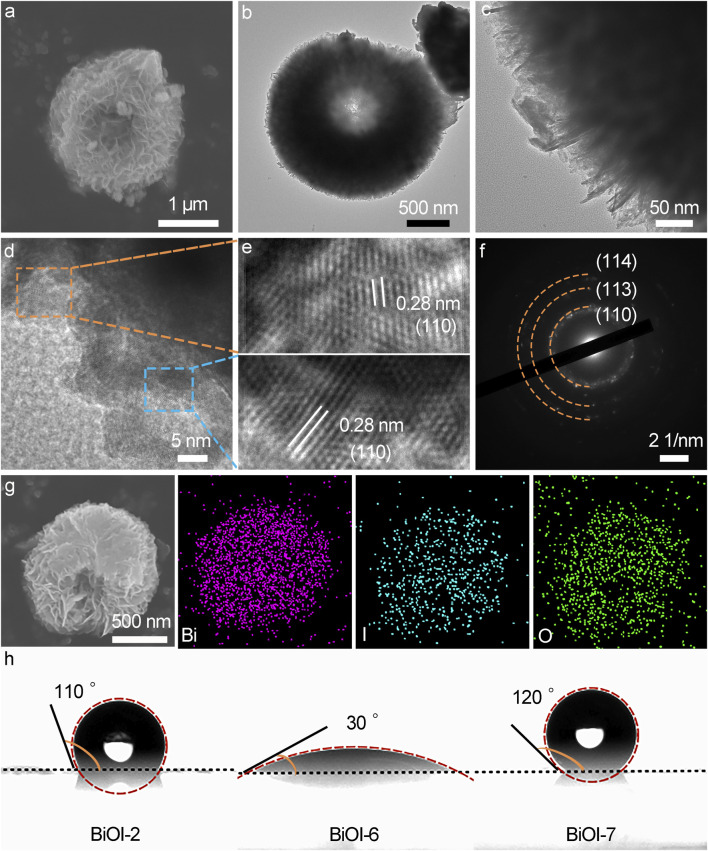
**(a)** SEM micrograph of BiOI-6. **(b,c)** TEM micrograph of BiOI-6. **(d,e)** HRTEM micrographs of BiOI-6. **(f)** SAED image of BiOI-6. **(g)** EDS elemental mappings of BiOI-6. **(h)** Water contact angles of BiOI-2, BiOI-6, and BiOI-7 samples.

Supplementary Figure S1 provides a comparative overview of the SEM morphologies for all synthesized samples from BiOI-1 to BiOI-9. The prepared BiOI photocatalyst consisted of numerous ultrathin nanosheets self-assembled into flower-like structures with a diameter of approximately 3 μm. These structures exhibited a large specific surface area and numerous voids, facilitating the transfer of charge carriers and the efficient separation of photogenerated electron–hole pairs (Norozi et al., 2024). Figure 2a corresponding to the SEM morphology of BiOI-6 sample with an, E.G.,/H_2_O volume ratio of 4:0, clearly show it hollow sphere structure with the smallest nanosheet thickness, which provided a higher number of reactive sites and enhanced the photocatalytic performance. The formation mechanism of BiOI hollow microspheres likely involves the self-assembly of nanosheets into intact flower-like spherical structures, followed by the removal of, E.G., during washing or drying, which creates an opening in the spherical structure. Supplementary Figure S1h shows the residual “lid” of the opening was the most favourable evidence. Supplementary Figure S1b correspond to the BiOI-2 sample with an, E.G.,/H_2_O volume ratio of 3:1. Compared to the samples discussed above, these catalysts exhibited a significantly increased particle size and nanosheet thickness. Supplementary Figure S1g, corresponding to the BiOI-7 sample with an, E.G.,/H_2_O volume ratio of 1:1, reveal that the nanosheet thickness of the microspheres was maximized, while their length was minimized. In summary, under the selected synthesis conditions among the three levels and three factors considered in the experiments, the solvothermal synthesis temperature and time had a minimal impact on the microstructure of BiOI samples, while the solvent type played a decisive role. This conclusion was consistent with the results of the orthogonal experiment. On synthesis mechanisms, when BiOI materials were synthesized using, E.G., as the solvent, Bi^3+^ cations could coordinate with, E.G., to form alcohol-salt complexes and release H^+^ ions, thus slowing down the I-Bi-O-Bi-I growth rate (Furukawa et al., 2024). The thickness of BiOI nanosheets and particle size of BiOI microspheres gradually decreased with increasing, E.G., content. And eventually the catalysts with controlled morphology could be synthesized by changing the concentration of solvent.

To further confirm the microstructure and exposed crystal planes on the surface of the BiOI samples, TEM and HRTEM analyses were conducted at various resolutions. Figures 2b,c show that the flower-like microspheres, which was formed through the self-assembly of BiOI-6 nanosheets, exhibited distinct hollow structures. Figures 2d,e reveals clear lattice fringes, and the 0.28 nm lattice spacing was attributed to the (110) crystal plane of BiOI. The SAED analysis in Figure 2f exhibits distinct diffraction rings corresponding to the (110), (113), and (114) lattice planes of the BiOI sample, confirming its polycrystalline nature.

To investigate the elemental composition and distribution of the BiOI catalyst, the sample was analyzed *via* EDS. The EDS results presented in Figure 2g reveal that the BiOI-6 catalyst consists of Bi, I, and O, which are uniformly distributed throughout the BiOI microspheres. Supplementary Figure S2 shows that the atomic percentages of Bi, I, and O were 17.48%, 17.44%, and 65.08%, respectively. This high oxygen content may be attributed to the oxygen-containing functional groups or water molecules adsorbed on the surface of the BiOI nanosheets.

The measurement of the water contact angle (WCA) is a commonly used method to evaluate the spreading of liquids on solid surfaces and determine the wettability of materials (Singhal and Shukla, 2020). A smaller contact angle indicates a greater spreading and higher wettability of the liquid on a solid surface (Li A. et al., 2024; Yabumoto et al., 2018). Figure 2h the water contact angles of the BiOI-2, BiOI-6, and BiOI-7 samples, while Supplementary Figure S3 provides a comparative analysis of the contact angles for the BiOI-1 to BiOI-9. Supplementary Figure S3f corresponds to the BiOI-6 sample. The images clearly show acute contact angles, with BiOI-6 exhibiting the minimum contact angle (approximately 30°) among all samples, indicating the highest surface hydrophilicity. This may be attributed to the high content of oxygen-containing functional groups or water adsorbed on the surface of the BiOI nanosheets, which directly improves the adsorption capacity and efficiency of the microspheres for pollutants in wastewater, thereby potentially promoting their removal under visible light irradiation. Supplementary Figure S3b correspond to the BiOI-2 sample; compared to the samples discussed above, they exhibited a significantly increased contact angle (∼110°). Supplementary Figure S3g shows the contact angles of the BiOI-7 sample, revealing that the contact angle further increased to approximately 120°. Overall, among the three levels and three factors selected in the experiments, the solvothermal synthesis temperature and time had a minimal effect on the contact angle of the BiOI samples, while the solvent type played a crucial role, consistent with the conclusions drawn from the orthogonal and XRD experiments. In the context of aqueous photocatalysis, high hydrophilicity is a crucial indicator as it facilitates the intimate contact between the catalyst surface and aqueous pollutants, which is an important prerequisite for efficient adsorption and subsequent surface redox reactions. Therefore, synthesizing a highly hydrophilic photocatalyst with high adsorption capacity is a key strategy for improving the wastewater treatment performance.

**FIGURE 3:**
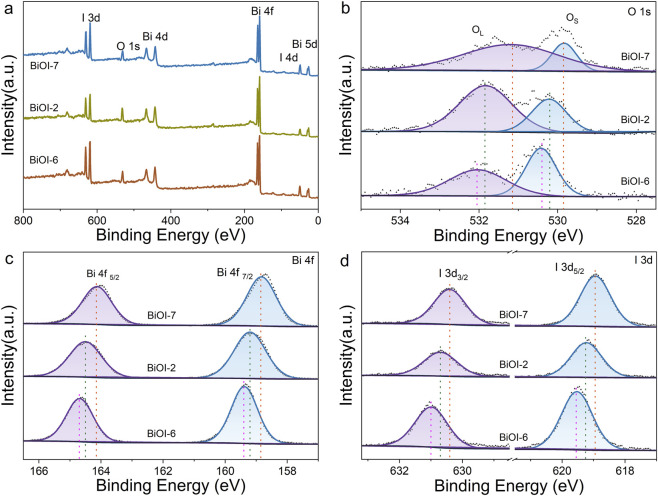
**(a)** XPS survey spectra. **(b)** O 1s, **(c)** Bi 4f, and **(d)** I 3d high-resolution spectra of BiOI-2, BiOI-6, and BiOI-7 samples.

XPS was used to analyze the chemical state of the BiOI-2, BiOI-6, and BiOI-7 samples. The XPS survey spectra shown in Figure 3a reveal the presence of Bi, O, and I on the surface, thus confirming the successful synthesis of the BiOI composite material. High-resolution XPS spectra were further obtained for O 1s, Bi 4f, and I 3d levels. Figure 3b shows two characteristic peaks at 529.85 and 531.15 eV, corresponding to lattice oxygen (O_L_) and hydroxyl groups or adsorbed H_2_O on the BiOI surface (O_S_), respectively (Abass et al., 2023). Notably, the areas of the O_S_ peak showed a progressive rise as, E.G., content increased, suggesting that the O_S_ species had a significant effect on the photocatalytic efficiency of the material. The photocatalytic efficiency increases with the proportion of O_S_ on the material surface. Additionally, the positions of the O_L_ and O_S_ peaks shifted toward higher binding energies, indicating a decrease in electron density, because the binding energy in XPS spectra is negatively correlated with the surface electron density (Bagus et al., 2023). Figure 3c shows the high-resolution XPS spectrum of Bi 4f levels, where the Bi 4f_5/2_ and Bi 4f_7/2_ peaks were located at binding energies of 164.15 and 158.85 eV, respectively, corresponding to Bi^3+^ (Tian et al., 2021). Figure 3d shows the high-resolution I 3d XPS spectrum, with the I 3d_3/2_ and I 3d_5/2_ peaks appearing at 630.4 and 618.95 eV, respectively, corresponding to I^−^ species (Zhang et al., 2021). With increasing, E.G., content, the Bi 4f and I 3d peaks gradually shifted toward higher binding energies, but their integral areas showed no significant change, indicating that the relative content of the corresponding elements was unaffected by the, E.G., content. This shift in binding energies can be attributed to surface complexation between, E.G., hydroxyl groups and Bi species on the BiOI surface.

The zeta potential can be used to assess the tendency of particles to aggregate, which reflects the stability of dispersed nanoparticles in solution (Won et al., 2023). In general, the higher the absolute value of the zeta potential, the more stable the particle dispersion system. The absolute value of the potential threshold for the dispersion stability of nanoparticles in the water phase is typically considered to be 20 mV (Khen et al., 2021). Above 20 mV, the dispersion is considered relatively stable. As shown in Supplementary Figure S4, the average absolute zeta potentials for the BiOI-7, BiOI-2, and BiOI-6 samples were 6.28, 21.39, and 34.20 mV, respectively, based on three repeated tests. The small error bars indicate that the test results were consistent. The absolute value of the zeta potential gradually increased as the, E.G., content increased, indicating an enhanced stability of the particle dispersion system in a water solution and a higher hydrophilicity. This result was in agreement with the results of the contact angle tests.

To verify the general nature of the excellent performance of the catalysts, the representative Rh B dye was selected as model pollutant for testing different samples under simulated visible light irradiation (Barveen et al., 2024). A beaker containing Rh B (10 mg/L [2.09 × 10^−5^ mol/L], 60 mL) contaminant solution and 20 mg (0.33 g/L) BiOI was placed under 22 °C circulating cooling water with magnetic stirring (500 r/min). The suspensions were stirred in the dark for 30 min to reach the adsorption–desorption equilibrium (dark adsorption) between the catalyst and the pollutant molecules (Jian et al., 2024). After visible light irradiation with a 300 W xenon lamp (equipped with a 420 nm cut-off filter to eliminate UV light), all samples were excited to produce photogenerated carriers (Yang et al., 2025). 4 mL of each sample were collected after a certain interval and centrifuged. Then, the supernatant was collected and examined by UV spectroscopy to determine the amount of contaminant degradation by the catalyst.

Further experiments were carried out to determine the photocatalytic degradation rate and the corresponding kinetic constants (Tang et al., 2021). The dye degradation rate and the first-order kinetic equation of photocatalytic removal for each sample were calculated according to Equations 1, 2:

$$\text{Dye degradation rate }\left(\%\right)=\left(C_{0}‐C\right)/C_{0}\times 100$$ (1)

$$‐\ln\left(\frac{C}{C_{0}}\right)=\text{kt}$$ (2)

where *C*_0_ is the initial concentration of the dye, *C* is the dye concentration at different irradiation times, and *k* is the rate constant of the first-order kinetic process (min^-1^).

**FIGURE 4:**
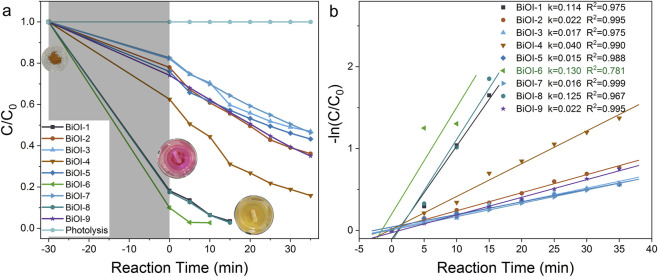
**(a)** Photodegradation of Rh B under visible light irradiation. **(b)** First-order kinetic plots of BiOI-1–BiOI-9 samples.

As shown in Figure 4a, the photolysis test confirmed that aqueous Rh B did not spontaneously undergo a photocatalytic reaction under visible light irradiation without adding any photocatalysts. The dark adsorption rates of the BiOI-3, BiOI-5, and BiOI-7 catalysts were only ∼20%; moreover, only ∼50% of RhB was removed after 35 min of visible light irradiation. In contrast, the BiOI-6 sample exhibited a dark adsorption rate of up to 90.1%, with a degradation rate of 99.7% after 10 min of visible light irradiation, demonstrating its excellent dark adsorption and extremely fast degradation rates. It is worth noting that BiOI-6 did not simply degrade the RhB dye through physical adsorption. As shown in the inset of Figure 4a, the yellow BiOI-6 powder sample turned pink after adsorbing the RhB dye and returned to its original color after degradation under visible light irradiation. This indicates that the dye removal process of BiOI-6 involved adsorption followed by degradation. As shown in Figure 4b, the *k* value of BiOI-6 (up to 0.130 min^-1^) was 8.7 times higher than those of the BiOI-5 and BiOI-7 samples, further demonstrating its superior degradation activity as photocatalyst.

**FIGURE 5:**
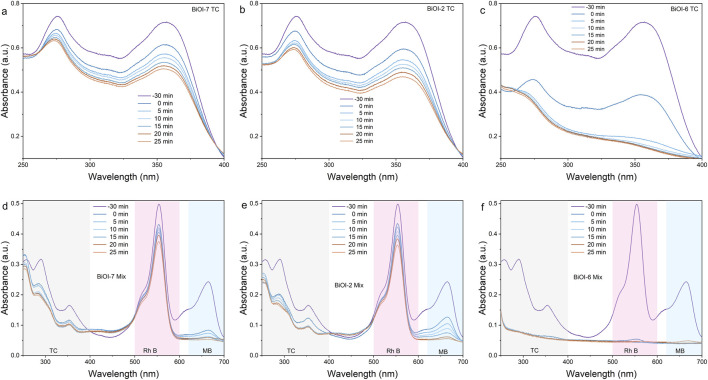
UV–vis absorption spectra of **(a)** BiOI-7/TC, **(b)** BiOI-2/TC, **(c)** BiOI-6/TC, **(d)** BiOI-7/mixed dyes (TC + Rh B + MB), **(e)** BiOI-2/mixed dyes, and **(f)** BiOI-6/mixed dyes at different irradiation times.

In order to mimic colorless pollutants and real polluted wastewater, Tetracycline (TC, 20 mg/L, 60 mL) and a mixture of three dyes (TC, Rh B, and Methylene blue [MB]) in equal volume ratios (10 mg/L, 20 mL each) were used to evaluate the degradation efficiencies of the catalysts. The UV–vis DRS absorption spectra of BiOI-7, BiOI-2, and BiOI-6 are shown in Figure 5. As displayed in Figures 5a,b, the dark adsorption efficiencies of the BiOI-7 and BiOI-2 catalysts for TC were 14.1% and 16.8%, and their photocatalytic degradation efficiencies after 25 min of irradiation were 29.4% and 34.3%, respectively. In comparison, as shown in Figure 5c, the BiOI-6 catalyst exhibited a dark adsorption efficiency of 45.9% for TC, and achieved a significant photocatalytic degradation rate of 78.9% after only 5 min of irradiation.

Further inspection of Figures 5d–f found no significant shift in the UV–vis absorption peaks of the mixed dye solution, strongly suggesting that the mixing process did not cause any significant changes in the physical and chemical properties of the mixed dyes. Similarly, the BiOI-7 and BiOI-2 catalysts showed relatively limited removal effects on the mixed dyes under dark adsorption and after 25 min of irradiation. However, the BiOI-6 catalyst again showed a superior performance, with the degradation rates for TC, Rh B, and MB reaching 60.8%, 89.0%, and 79.8% under dark adsorption and further increasing to 66.9%, 91.0%, and 83.5% after 5 min of irradiation, respectively. In summary, the BiOI-6 catalyst exhibited an excellent dark adsorption capacity and an extremely fast photocatalytic degradation rate for both TC and the mixed dye solution. As shown in Supplementary Table S2, some of the BiOI-based catalysts reported in the literature were compared with BiOI photocatalysts in this work, and it can be found that flower-like BiOI-6 hollow microspheres exhibit excellent photocatalytic degradation performance for Rh B and TC, and therefore has a promising application prospect in wastewater treatment.

**FIGURE 6:**
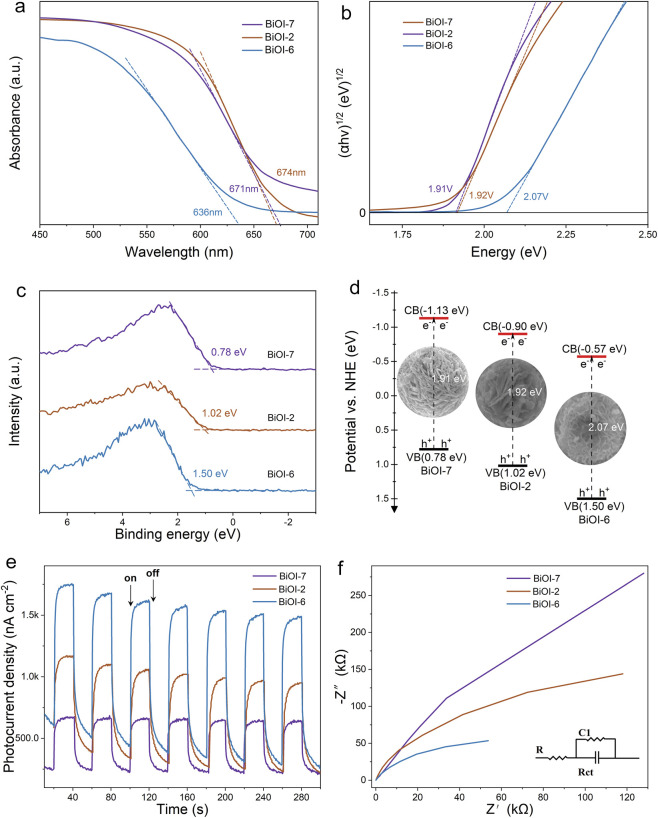
**(a)** UV−vis DRS spectra, **(b)** Tauc plots, **(c)** XPS valence band spectra, **(d)** Energy band diagrams, **(e)** Transient photocurrent responses and corresponding **(f)** EIS Nyquist plots of BiOI-2, BiOI-6, and BiOI-7.

UV–vis DRS was employed to investigate the optical absorption characteristics of the catalysts (Yang Z. et al., 2022). As shown in Figure 6a, the absorption edges of BiOI-2, BiOI-6, and BiOI-7 were located at 671, 636, and 674 nm, respectively. This indicated that the photocatalysts exhibited an excellent absorption capacity for visible light, highlighting their potential for effective photocatalytic applications.

The bandgap energy (Eg) of each sample was estimated using the Kubelka–Munk Equation 3:

$${\left(\alpha hv\right)}^{1/n}=A\left(hv-E_{g}\right)$$ (3)

where *α* denotes the absorption coefficient obtained from UV–vis DRS, *h* is Planck’s constant, *υ* is the optical frequency, and *A* is a constant; moreover, BiOI is an indirect bandgap semiconductor, *n* = 2 (Du et al., 2023; Makuła et al., 2018). As shown in Figure 6b, the *E*_g_ values of BiOI-2, BiOI-6, and BiOI-7 were calculated to be 1.92, 2.07, and 1.91 eV, respectively.

In addition, the valence band (VB) positions of different samples were determined by analyzing the XPS valence band spectra. As shown in Figure 6c, the VB positions of BiOI-2, BiOI-6, and BiOI-7 were 1.02, 1.50, and 0.78 eV, respectively. The conduction band (CB) position was obtained using the following Equation 4:

$$E_{\text{CB}}=E_{\text{VB}}‐E_{g}$$ (4)

Therefore, the CB positions of BiOI-2, BiOI-6, and BiOI-7 were −0.90, −0.57, and −1.13 eV, respectively, confirming the bandgap structure of BiOI. A more negative *E*_CB_ is known to reflect a higher reducing capacity; conversely, a more positive *E*_VB_ indicates a higher oxidizing capacity. Therefore, the above results show that BiOI-7 and BiOI-6 had the highest reducing and oxidizing abilities in Figure 6d, respectively.

To further investigate the migration and separation rates of photogenerated e^−^/h^+^ pairs, transient photocurrent responses and EIS data were obtained for the BiOI samples. As shown in Figure 6e, all samples exhibited significant photocurrent responses upon switching on light irradiation. Among them, BiOI-6 exhibited the strongest photocurrent response, indicating that the optical carriers generated by this catalyst under irradiation conditions had the highest transmission efficiency.

Furthermore, the charge transfer resistances of the prepared catalysts were investigated using EIS. A smaller arc radius in the Nyquist plot indicates a higher photogenerated carrier separation rate and a lower charge transfer resistance (Bao et al., 2023). As shown in Figure 6f, among the investigated samples, the Nyquist curve of BiOI-6 exhibited the smallest arc radius, indicating that BiOI-6 had a lower charge transfer resistance than the other samples. The highest carrier transport efficiency and lowest charge transfer resistance of BiOI-6 reflect its superior photocatalytic efficiency. The conclusions obtained on the basis of the photocurrent response and EIS data confirm that BiOI-6 exhibited the best photocatalytic efficiency; this can be attributed to the gradual decrease in the thickness of the BiOI nanosheets with increasing amount of, E.G., which improved the migration and separation efficiency of photogenerated e^−^/h^+^ pairs.

**FIGURE 7:**
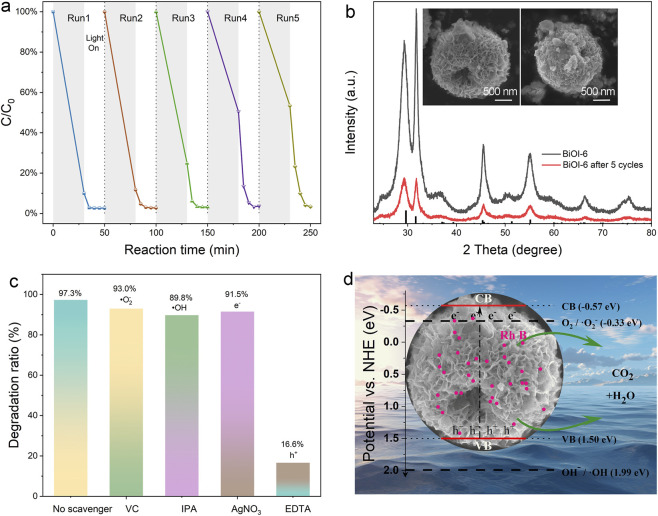
**(a)** Recycling stability test, **(b)** XRD patterns, (insets in b) SEM micrographs before and after five catalytic cycles, **(c)** Active species trapping experiments and **(d)** Schematic illustration of photocatalytic reaction mechanism of BiOI-6.

In order to assess the cycling stability of the catalyst, the BiOI-6 sample was recovered by centrifugation after each photocatalytic removal experiment and used in subsequent tests. The photocatalytic degradation rates are shown in Figure 7a. After five cycles, the dark adsorption rate gradually decreased from 90% (in the first cycle) to 50%. The comparison of the SEM micrographs before and after cycling (insets in Figure 7b) suggests that the possible reason for this decrease was that the sheet-like structure of the BiOI nanosheets was destroyed during the recovery process; the detached nanosheets were difficult to recover and easily aggregated covering the surface of the microspheres, reducing their specific surface area, and directly affecting their adsorption rate for Rh B. Figure 7b shows the XRD patterns before and after five cycles; all the obvious diffraction peaks corresponded to the standard JCPDS card No. 10-0445, indicating that the BiOI phase remained unchanged after cycling. However, the intensities of all diffraction peaks decreased after fifth cycle, indicating a lower crystallinity of the sample. This may also be due to the destruction of the sheet-like structure of the BiOI-6 surface nanosheets during the recovery process. Nevertheless, the BiOI-6 catalyst still exhibited excellent photocatalytic degradation efficiency with a degradation rate of more than 96.1% in the fifth cycle under visible light irradiation within 20 min. Comparing the fresh catalyst with the sample after the fifth cycle, Supplementary Figure S5a shows the characteristic FTIR absorption bands, notably peaks at around 1,590 cm^-1^ and 3,440 cm^-1^ can be assigned to the bond-stretching vibrations of O-H because of the adsorption of water molecules or the hydroxyl groups on the catalyst surface (Benisti and Paz, 2019), and peaks below 1,000 cm^-1^ are attributed to Bi-O bond-stretching vibrations (Yadav et al., 2020), while the peak at 1,300 cm^-1^ is due to the stretching vibrations of the tetragonal crystal links of BiOI (Mera et al., 2017), all these spectral features remain at nearly unchanged positions across the cycles. Supplementary Figure S5b shows that no significant changes were observed in the Raman spectrum before and after 5 cycles. The observed, E.g., and A_1_g modes are associated with the external vibration of iodine atoms and the stretching vibration of Bi-O bonds within the [Bi_2_O_2_]^2+^ layers, respectively (Yang et al., 2024). Crucially, no new peaks appear in the used sample’s spectrum indicates that organic pollutants or reaction intermediates were not adsorbed onto the catalyst surface. This result confirmed that the photocatalyst exhibits an excellent stable structure, which is an important requirement for its application in polluted water environments.

We next studied the capture of different radicals under visible light to identify the main active species in the photocatalytic reaction process and reveal the potential reaction mechanism of the BiOI-6 material. VC, IPA, AgNO_3_, and EDTA-2Na were used as trapping agents for superoxide radical anions (·O^2−^), hydroxyl radicals (·OH), e^−^, and h^+^, respectively (Yang S. et al., 2022). The trapping agent was added to the Rh B solution at a fixed ratio before the dark treatment. The resulting degradation efficiencies are shown in Figure 7c. Using EDTA -2Na as trapping agent, the Rh B degradation rate of BiOI-6 after visible light irradiation for 10 min was only 16.6%. When VC, IPA, and AgNO_3_ were used as trapping agents, the degradation rate reached 93.0%, 89.8%, and 91.5%, respectively. Compared to a Rh B degradation rate of 97.3% without trapping agents, the addition of EDTA-2Na significantly inhibited the photocatalytic degradation of Rh B. Conversely, VC, IPA, and AgNO_3_ had little effect on the removal reactions. This indicates that h^+^ species played the most important role in the photocatalytic process, whereas O^2−^, ·OH, and e^−^ were not the main active particles.

A potential mechanism for the photogenerated carrier production, separation, transfer, and photocatalytic destruction of the BiOI materials was proposed based on the above findings. First, the standard redox potential *E*_θ_ of the OH^−^/OH couple (1.99 eV vs. NHE) was more positive than the *E*_VB_ value (1.50 eV vs. NHE) of BiOI-6; therefore, the catalyst could not oxidize OH^−^ to OH. Second, h^+^ played the most important role in the photocatalytic process, and BiOI-6 had the most positive *E*_VB_ and the highest oxidative capacity. Therefore, among the BiOI samples investigated in this study, BiOI-6 demonstrated superior photocatalytic degradation performances for Rh B as displayed in Figure 7d. Finally, we provide a summary of the photocatalytic process of the samples, which involved the following reactions (Equations 5–7):

$$\text{BiOI}+\text{hv}\left(i.e.,\text{visible light}\right)\to h^{+}+e^{‐}$$ (5)

$$O_{2}+e^{‐}\to \cdot O_{2}^{‐}$$ (6)

$$h^{+}\text{ or }\cdot O_{2}^{‐}\text{ or }e^{‐}+\text{pollutant}\to {\text{CO}}_{2}+H_{2}O+\text{intermediate products}$$ (7)

## 4 Conclusion

In this study, the solvothermal method based on an orthogonal experimental design was employed to prepare BiOI nanoparticles with excellent photocatalytic activity. The effects of different solvent ratios, reaction times, and temperatures on the degradation efficiency of organic pollutants were investigated, and the optimal preparation conditions for the BiOI catalyst were identified. Flower-like BiOI-6 hollow microspheres were prepared with ethylene glycol as a solvent at a reaction temperature of 120 °C, achieving a degradation rate of 97.3% after 10 min of irradiation for Rh B. The BiOI-6 photocatalyst exhibited excellent dark adsorption and ultrafast photocatalytic degradation rates. Free radical trapping experiments indicated that h^+^ was the main active species. This study explored the internal relationship between microstructure and photocatalytic efficiency of BiOI materials, revealing the enhancement mechanism of the photocatalytic efficiency, as well as elucidating the charge transfer path and degradation mechanism of pollutants in the photocatalytic system. Finally, a series of analytical tests demonstrated that the solvent significantly affected the photocatalytic efficiency of the catalyst by modifying its microscopic morphology. This research is thought to provide a new direction for constructing highly efficient photocatalysts to address energy conversion and environmental pollution challenges.

## Data Availability

The raw data supporting the conclusions of this article will be made available by the authors, without undue reservation.
