# Supplementary material for: Tailoring the architecture of BiOI microspheres for enhanced photocatalytic wastewater purification
Source: Front Chem. 2026 May 29;14:1833474. doi: 10.3389/fchem.2026.1833474 (PMC13260337; doi:10.3389/fchem.2026.1833474)
Supplement: Supplementary file 1 [file DataSheet1.docx]

**Supporting Information**

**Tailoring the Architecture of BiOI Microspheres for Enhanced Photocatalytic Wastewater Purification**

Yongxiang Ma ^1,2^, Junzhi Li ^2^, Shujie Liu ^2^, Liang Qiao ^2^, Xiuhua Zhu ^1^, Zhihua Zhang ^1,^^*^, Xiaoying Hu ^2,*^

^1^ School of Materials Science and Engineering, Dalian Jiaotong University, Dalian 116028, China

^2^ School of Materials Science and Engineering, Key Laboratory of Materials Design and Quantum Simulation, Changchun University, Changchun 130022, China.

* Corresponding author:

Zhihua Zhang, zhzhang@djtu.edu.cn; Xiaoying Hu, huxy@ccu.edu.cn

**Experimental section**

***Characterization:*** The crystal structures of the samples were determined by X-ray powder diffraction (XRD, Bruker D8 Advance) using a Cu Kα radiation source (*λ* = 0.15418 nm). The surface topographies and microstructures of the samples were investigated by scanning electron microscopy (SEM, Hitachi SU8010), energy dispersive X-ray spectroscopy (EDS), transmission electron microscopy (TEM, JEOL JEM-2100F), high-resolution TEM (HRTEM, JEOL JEM-2100F), and selected-area electron diffraction (SAED, JEOL JEM-2100F). X-ray photoelectron spectroscopy (XPS, ESCALAB 250) was employed to analyze the molecular structures, chemical states, and elemental compositions of the samples. An ultraviolet–visible (UV–vis) spectrometer (Shimadzu UV2500) was used to obtain UV–vis diffuse reflectance spectroscopy (DRS) profiles of the dye solutions degraded by the catalysts after different irradiation times, as well as to monitor the concentration changes of the supernatants, in order to evaluate the photocatalytic efficiencies of the catalysts. The measured zeta potentials (Malvern Panalytical, DTS1070) were used to assess the particle aggregation tendency, reflecting the stability of dispersed nanoparticles. Contact angle measurements (Dataphysics Contact Angle System, OCA 25) are widely used for evaluating the dispersion of a liquid on solid substrates and for assessing the wettability of a material. Fourier transform infrared (FTIR) spectra were recorded on a Shimadzu IRTracer-100 spectrometer. The Raman spectra were acquired using a HORIBA LabRAM Odyssey confocal micro-Raman spectrometer equipped with a 532 nm laser.

***Electrochemical measurements:*** An electrochemical workstation (Shanghai Chenhua, CHI660E) with a three-electrode system was used to investigate the photocatalytic efficiencies of the samples. The transient photocurrent responses and electrochemical impedance spectroscopy (EIS) data were also obtained and analyzed. Prior to the test, 1 mg of BiOI powder was mixed with 0.2 mL of ethanol and 0.3 mL of deionized water. After ultrasonic stirring for 30 min, the solution was evenly spread on a 1 × 1 cm conductive glass substrate (FTO) to dry and form a film. The sample was then mounted on an electrode holder as the working electrode, with silver/silver (Ag/AgCl) chloride as the reference electrode, platinum as the counter electrode, and a 1 M sodium sulfate solution as the electrolyte. The test was performed in a quartz electrolysis cell with a 300 W xenon lamp (PLS-SXE300) equipped with a 420 nm filter as the visible light source.

***Wettability measurements:*** Before conducting electrochemical measurements on the FTO film samples, wettability measurements were evaluated using a Dataphysics contact angle system. Mount the film sample on a clean, flat, and level stage. Carefully dispense a 1μL droplet of deionized water onto the surface using a microsyringe. Allow the droplet to stabilize for at least 30 seconds to reach equilibrium. Capture a side-view image of the droplet using a high-resolution digital camera equipped with a zoom lens and backlight illumination. Analyze the droplet profile using software included with the device to determine the water contact angle.


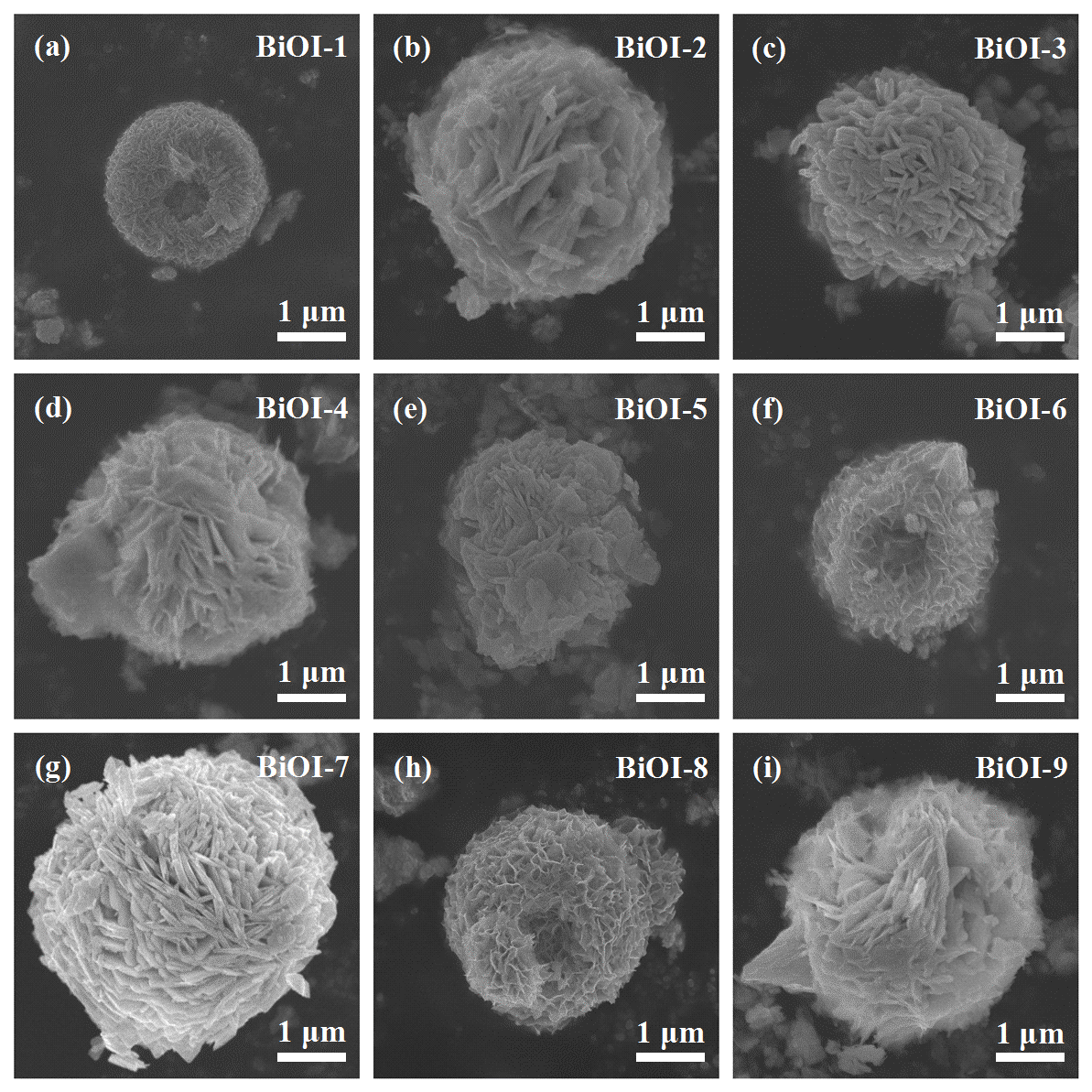


**Fig. S1.** SEM micrographs of BiOI-1–BiOI-9 samples.

**
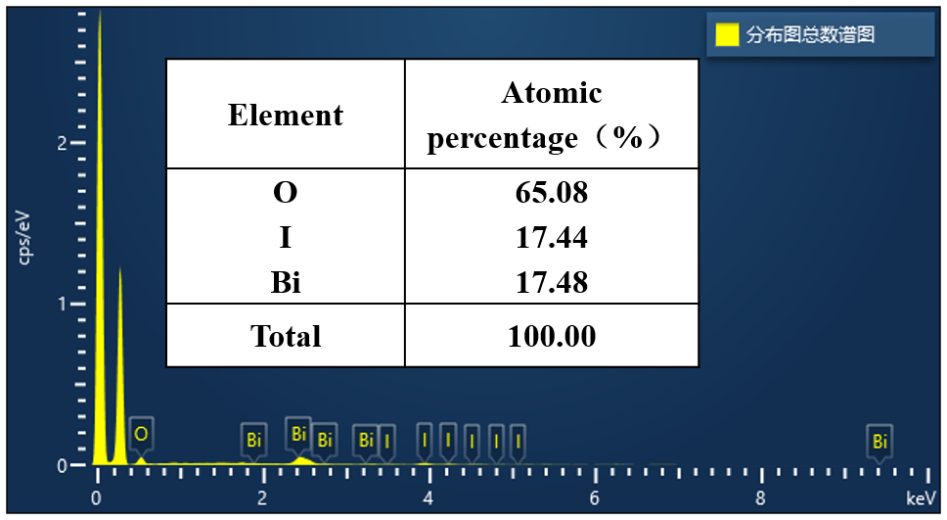
**

**Fig. S2.** EDS spectrum of BiOI-6 sample.


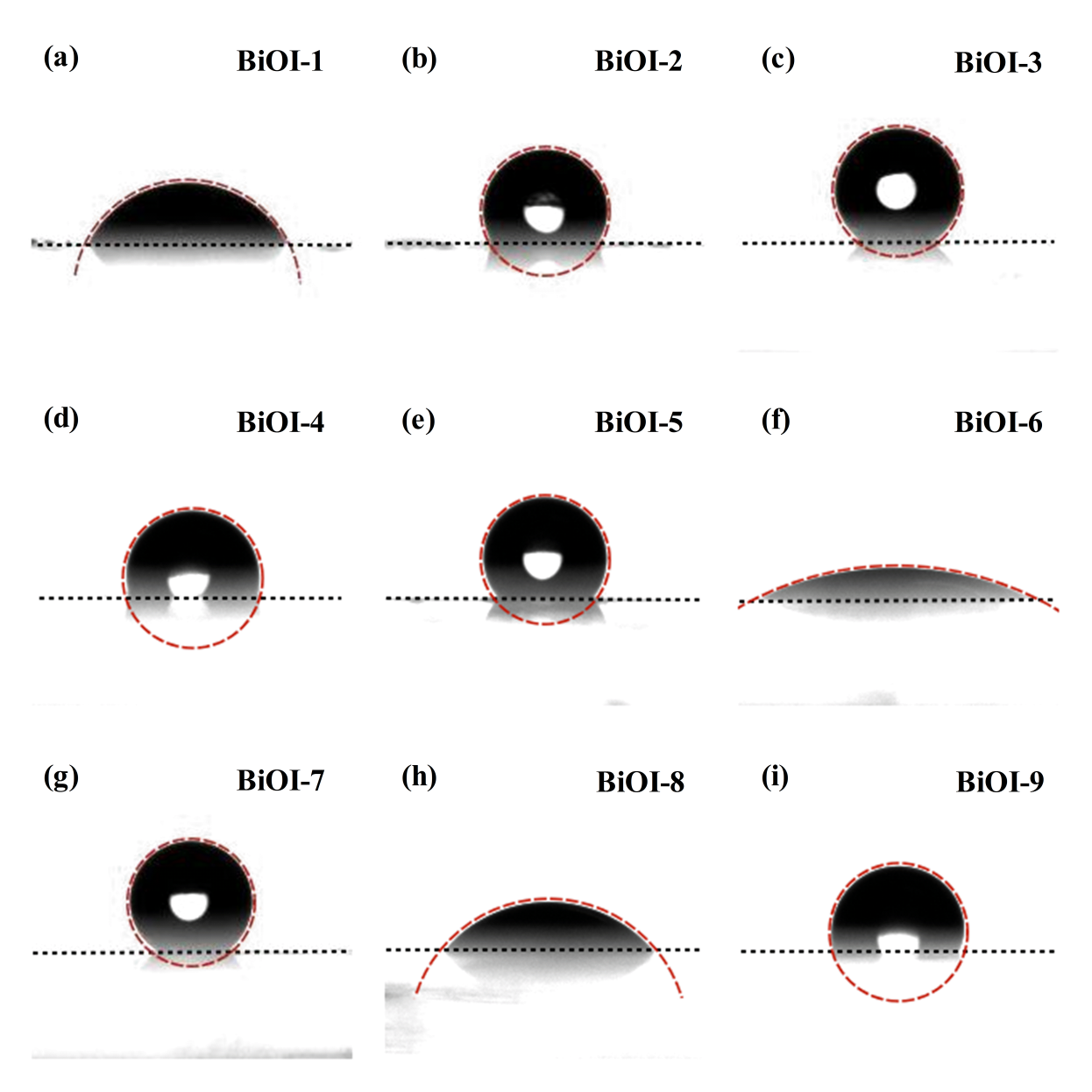


**Fig. S3.** Water contact angles of BiOI-1–BiOI-9 samples.


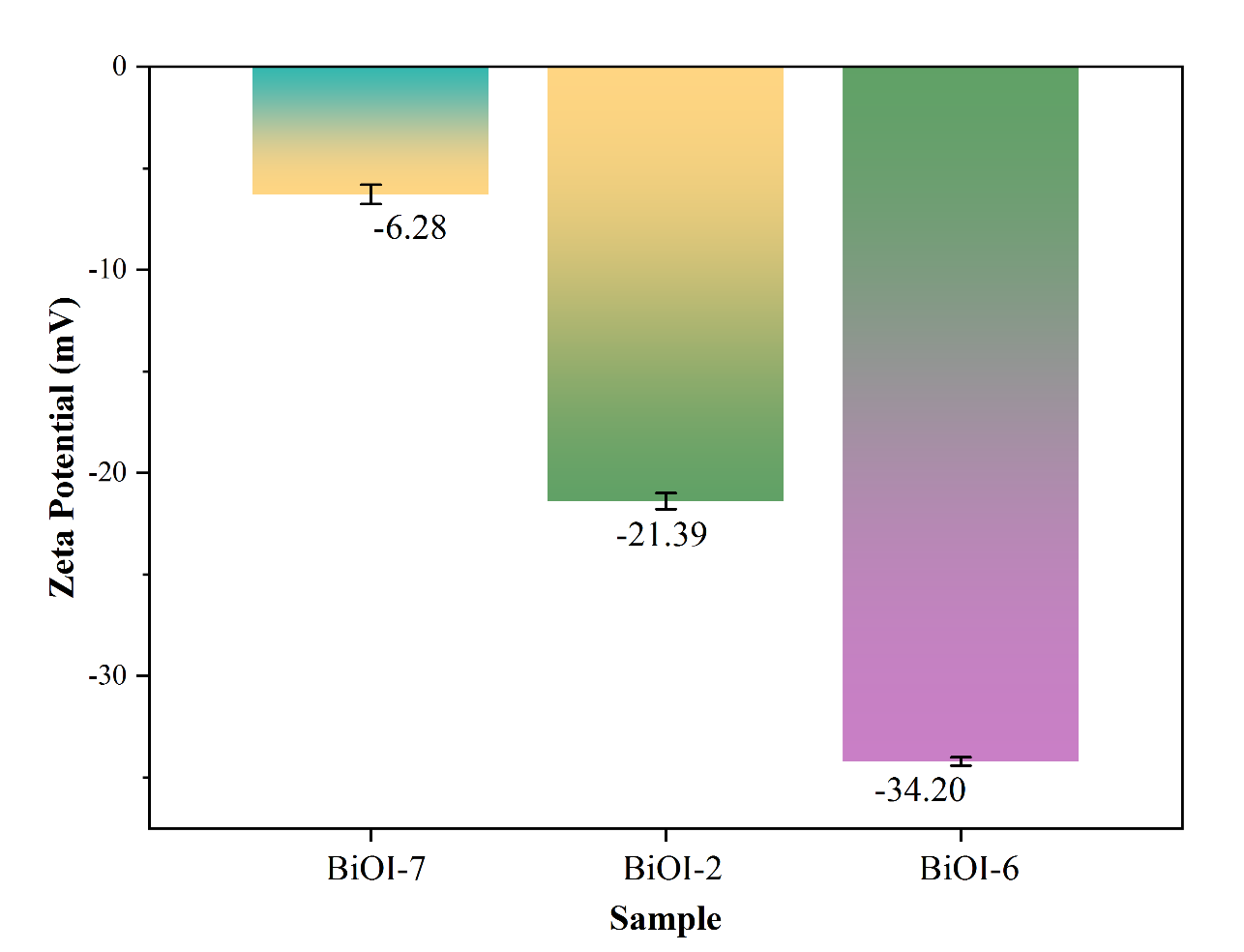


**Fig. S4.** Zeta potentials of BiOI-7, BiOI-2, and BiOI-6.

**
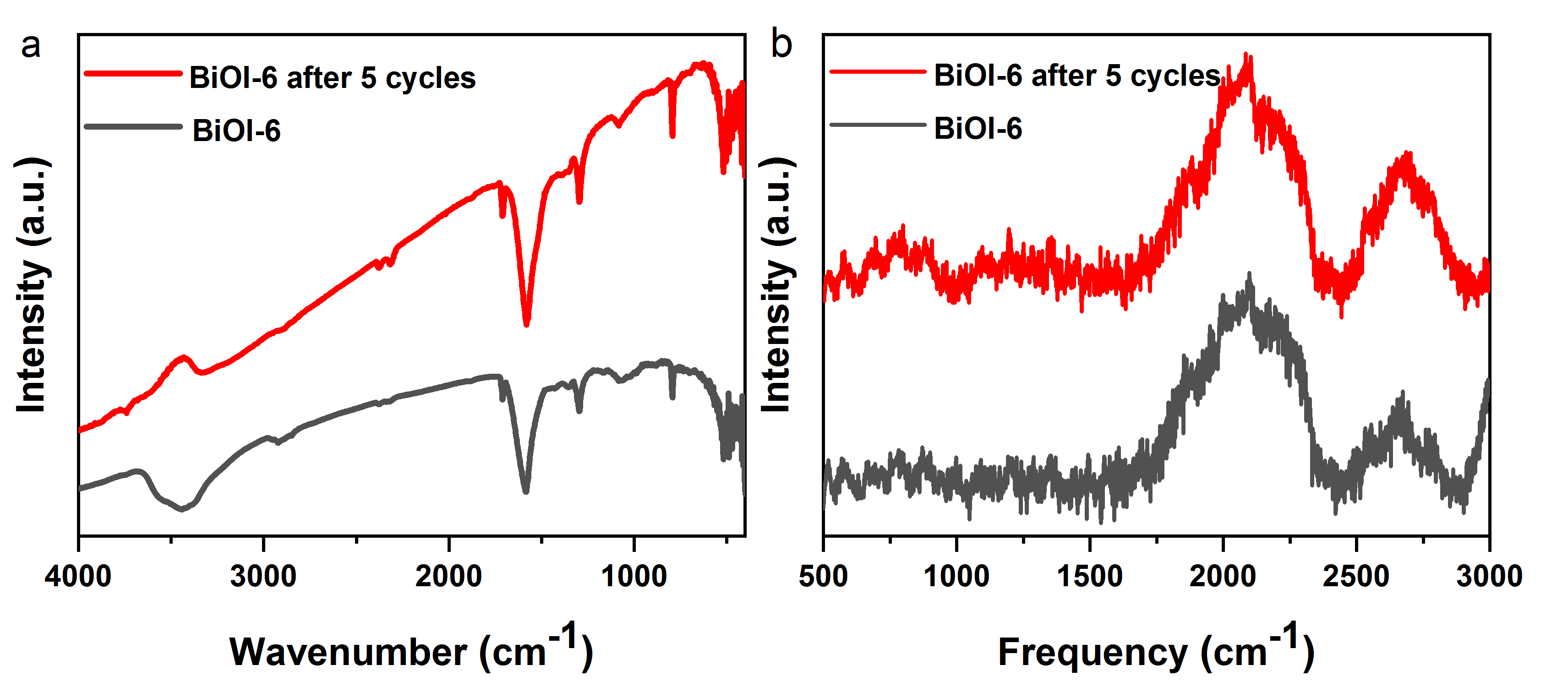
**

**Fig. S5.** (a) FTIR (b) Raman spectrum before and after five catalytic cycles of BiOI-6.

**Table S1** Levels of each factor in orthogonal experiment.

| **Factor** | | **Level** | | |
| --- | --- | --- | --- | --- |
|  |  | **1** | **2** | **3** |
| A | Temperature (°C) | 100 | 120 | 140 |
| B | Time (h) | 6 | 8 | 10 |
| C | Volume ratio of EG to H_2_O | 4:0 | 3:1 | 1:1 |
| D | Control group |  |  |  |

**Table S2** Comparison of degradationl performance by the different photocatalysts.

| **Photocatalytic system** | **Simulated pollutants** | **Construction of dye and catalyst amount** | **Reaction condition** | **Degrada-**  **tion rate** | **Reference** |
| --- | --- | --- | --- | --- | --- |
| Bi_3_O_4_Cl/BiOI | Rh B | 10mg/L 300mL 100mg | 500W 90min | 85% | S(Cheng et al., 2020) |
| Bi_2_WO_6_/BiOBr  Bi_2_WO_6_/BiOCl  Bi_2_WO_6_/BiOI | Rh B | 10mg/L 45mL 45mg | 55W 8min | 98%  65%  26% | S(Derikvand et al., 2021) |
| MIL-53(Fe)/BiOI | TC | 25mg/L 50mL 50mg | 300W 14min | 86.2% | S(Ma et al., 2021) |
| Bi_4_O_5_I_2_/BiOI | Rh B  TC | 10mg/L 50mL 50mg | 500W 120min | 82%  62% | S(Lai et al., 2023) |
| g-C_3_N_4_/BiOI | TC | 20mg/L 100mL 40mg | 500W 60min | 84.2% | S(Luo et al., 2024) |
| CoTiO_3_/BiOI | Rh B  TC | 10mg/L 100mL 30mg | 500W 60min  500W 80min | 97%  74.8% | S(Lu et al., 2022) |
| BiOI | Rh B  TC | 20mg/L 60mL 20mg | 300W 5min | 99.7%  78.9% | This work |

**Reference**

1. Cheng L., Xiao X., Wang Y.,Lu M. (2020). Fabrication of microsphere-like Bi_3_O_4_Cl/BiOI Z-scheme heterostructure composites and its enhanced photocatalytic performance for degradation of MO and RhB. *Res. Chem. Intermed.*, 46(10), 4685-4704. doi: 10.1007/s11164-020-04231-7

2. Derikvand L.,Tahmasebi N. (2021). Synthesis and photocatalytic performance of Bi_2_WO_6_/BiOX (X=Cl, Br, I) composites for RhB degradation under visible light. *Korean J. Chem. Eng.*, 38(1), 163-169. doi: 10.1007/s11814-020-0687-y

3. Ma Y., Li M., Jiang J., Li T., Wang X., Song Y., et al. (2021). In-situ prepared MIL-53(Fe)/BiOI photocatalyst for efficient degradation of tetracycline under visible-light driven photo-Fenton system: Investigation of performance and mechanism. *J. Alloys Compd.*, 870, 159524. doi: 10.1016/j.jallcom.2021.159524

4. Lai C., Luo L., Chen Y., Chen J.,Zhong J. (2023). In-situ construction of S-scheme Bi_4_O_5_I_2_/BiOI heterojunctions with enriched oxygen vacancies and enhanced photocatalytic properties towards destruction of rhodamine B and tetracycline. *Inorg. Chem. Commun.*, 158, 111622. doi: 10.1016/j.inoche.2023.111622

5. Luo X., Pu S., Duan Y., Mao L., Lei K.,Sun Y. (2024). Facile construction of Z-scheme g-C_3_N_4_/BiOI heterojunction for improving degradation of tetracycline antibiotics. *Mater. Lett.*, 354, 135408. doi: 10.1016/j.matlet.2023.135408

6. Lu X., Wang L., Li Q., Luo R., Zhang J.,Tian Z. (2022). Construction of CoTiO_3_/BiOI p-n heterojunction with nanosheets-on microrods structure for enhanced photocatalytic degradation of organic pollutions. *Adv. Powder Technol.*, 33(1), 103354. doi: 10.1016/j.apt.2021.11.006
